# Facial Expression Time Processing in Typical Development and in Patients with Congenital Facial Palsy

**DOI:** 10.3390/brainsci12050516

**Published:** 2022-04-19

**Authors:** Mauro Belluardo, Elisa De Stefani, Anna Barbot, Bernardo Bianchi, Cecilia Zannoni, Alberto Ferrari, Holly Rayson, Santo Di Nuovo, Giovanni Belluardo, Paola Sessa, Pier Francesco Ferrari

**Affiliations:** 1 Unit of Neuroscience, Department of Medicine and Surgery, University of Parma, 43125 Parma, Italy; elidestefani@gmail.com (E.D.S.); alibi.ferrari@gmail.com (A.F.); 2 Unit of Paediatric Neuropsychiatry, Reggio Emilia Hospital, 42019 Scandiano, Italy; 3 Unit of Audiology and Paediatric Otorhinolaryngology, University Hospital of Parma, 43125 Parma, Italy; annabarbot59@icloud.com; 4 Maxillo-Facial Surgery Operative Unit, Head and Neck Department, University of Parma, 43125 Parma, Italy; bbianchi@ao.pr.it (B.B.); ceci.zannoni@gmail.com (C.Z.); 5 Institut des Sciences Cognitives Marc Jeannerod, CNRS/Université Claude Bernard Lyon, 69675 Bron, France; holly.rayson@isc.cnrs.fr; 6 Department of Educational Sciences, University of Catania, 95124 Catania, Italy; sdinuovo@unict.it; 7 Italian Association of Psychology (AIP), 00186 Rome, Italy; 8 Department of Political Sciences, University of Catania, 95131 Catania, Italy; gbellua@unict.it; 9 EGLE Institute of Psychology and Psychotherapy, 95131 Catania, Italy; 10 Department of Developmental Psychology and Socialization, University of Padova, 35131 Padova, Italy; paola.sessa@unipd.it

**Keywords:** facial palsy, emotion, facial expressions, time processing, social interactions, emotional and social development

## Abstract

Temporal dynamics of behavior, particularly facial expressions, are fundamental for communication between individuals from very early in development. Facial expression processing has been widely demonstrated to involve embodied simulative processes mediated by the motor system. Such processes may be impaired in patients with congenital facial palsy, including those affected by Moebius syndrome (MBS). The aims of this study were to investigate (a) the role of motor mechanisms in the processing of dynamic facial expression timing by testing patients affected by congenital facial palsy and (b) age-dependent effects on such processing. Accordingly, we recruited 38 typically developing individuals and 15 individuals with MBS, ranging in age from childhood to adulthood. We used a time comparison task where participants were asked to identify which one of two dynamic facial expressions was faster. Results showed that MBS individuals performed worse than controls in correctly estimating the duration of facial expressions. Interestingly, we did not find any performance differences in relation to age. These findings provide further evidence for the involvement of the motor system in processing facial expression duration and suggest that a sensorimotor matching mechanism may contribute to such timing perception from childhood.

## 1. Introduction—Hic et Nunc, Together: Mechanisms of Temporal Processing, from Pure Time Processing to Synchronous Interaction

From the first theorization of ancient Greek philosophers to more recent phenomenological and psychological conceptualizations [1,2], time has been considered a fundamental dimension of the human experience. The ancient Greeks, in addition to “Chronos”, the objective and sequential time, and “Ayon”, the eternal time, defined “Kairos” as the right and opportune moment in which something special should happen—in other words, a propitious moment to act and to be seized. Indeed, time is a crucial kinematic feature of action, defining fundamental aspects of behavior. From an ethological and evolutionary perspective, it is particularly adaptive in the animal domain to produce an action with a specific duration/speed, in a specific moment (e.g., attack or flee in the presence of a threat, or act together with in-group members in order to cooperate). In this respect, time can be considered an intrinsic relational dimension at the foundation of individual–environment interactions.

Several ethological and behavioral investigations have revealed that animals can synchronize their behavior and endogenous rhythms with external events (a phenomenon called entrainment), both in relation to physical (e.g., specific moments of the day and night or seasons of the year) and social environmental features. In fact, behavioral synchrony between individuals is fundamental for social interaction, both for human and non-human primates, allowing for reciprocal emotion regulation, the fostering of affiliation, and social organization [3,4]. Estimating the timing of behaviors performed by others is a critical aspect in promoting functional interactions, not only in specific domains such as music or sport, but in everyday social contexts (e.g., extending your hand to shake at the right time as someone else is giving you their hand, smiling in response to another’s smile, respecting reciprocal turn-taking during a conversation), and even during early mother–infant interactions [5]. Indeed, social interactions are characterized by reciprocal affective “attunement”. This primarily involves bodily and non-verbal aspects of communication, in particular, facial expressions of emotions [6,7,8,9,10] in which temporal dynamics such as reciprocal synchronicity represent a core aspect [2,5]. Therefore, it can be hypothesized that a biologically predisposed mechanism exists that underlies correct estimation of observed action/facial expression timing, with the transfer of this information into one’s own motor programs and behavior enabling the production of a coherent and adequate action/facial expression in response to the observed one. From a neurobiological perspective, a highly supported model posits the existence of a distributed network for facial expression processing [11]. This network comprises visual areas of the temporal cortex, including the posterior superior temporal sulcus (pSTS), as well as motor regions. The pSTS is primarily engaged by biological motion, and motor regions contain maps of facial expressions [12] linked to the mirror neuron system (MNS), which is active both during the production and the observation of actions and facial expressions [13,14,15,16,17]. Brain imaging studies in humans have also shown that the observation and imitation of emotional facial expressions activate a network of regions in addition to premotor and somatosensory parietal regions, including specific limbic structures (i.e., the anterior insula, the amygdala, and the anterior cingulate cortex). These limbic structures are involved in the modulation of autonomic and vegetative responses coupled with expressions of emotion [12,13,17,18,19,20,21]. Notably, several simulation models [10,22] propose that the motor system is crucial for emotion processing by automatically simulating the observed emotion (both in terms of autonomic activity and behavior) based on one’s own motor representations. However, to date, it is unknown if the motor system contributes not only to action/emotion recognition, but also to the temporal processing of facial expressions, or if this specific process is based predominantly on neural and cognitive processes other than a sensorimotor mechanism. As such, one of the main objectives of the present work is to further clarify the role of such motor mechanisms in processing the timing of dynamic facial expressions.

Classical models of time perception focus largely on the ability to process “pure” time, both in terms of the production of simple motor acts (e.g., finger tapping) and the processing of neutral visual and/or auditory perceptual stimuli. From a developmental point of view, previous research has also explored “pure” time processing abilities in both adults and children, demonstrating the existence of time processing competence from early on in life which then improves with age [23].

One of the most accredited cognitive models of time processing is that of the “internal clock” [24,25,26]. This model posits the existence of a biologically based mechanism that allows animals and humans to process time using an inner clock composed of three main elements: the switch, which closes at the beginning and opens at the end of an event to be timed; an inner pacemaker, which emits pulses; and an accumulator, which keeps track of time through the accumulation of pulses. A sense of time and estimation of duration are posited to arise from the number of pulses accumulated by the accumulator from the beginning (closing of the switch) until the end (opening of the switch) of an event. Although findings are not conclusive, to date considerable evidence has been provided for one neurobiological model of the internal clock, the striatal beat frequency model (SBF) [27,28]. According to this model, based on the specificity of the stimulus to be timed, large-scale cortical activity is characterized by a specific dopaminergic modulation of oscillatory patterns/beats, which, in turn, are detected by spiny neurons of the striatum through downstream cortico-subcortical projections. In keeping with this neurobiological model, meta-analyses [29,30,31,32] indicate the existence of a specific cortico-subcortical neural network for time processing, which depends on the duration of the stimuli (supra-second or sub-second) as well as on the type of task (perceptual or motor). In addition these studies also posited [29,30,31,32] that the core network revealed for time processing includes two crucial regions of the motor system: the supplementary motor area (SMA) and the inferior frontal gyrus (IFG). Interestingly, these regions are always active during both perceptual and motor time processing tasks, regardless of the duration of the stimuli.

To date, few studies have investigated if similar cognitive and neurobiological mechanisms are related not only to the processing of “pure” time stimuli, but also to the temporal processing of complex biological motion across development, in particular, stimuli that have affective value.

A recent study by Allingham and colleagues [33] investigated adults’ ability to process the timing of biological dance-like movement, showing that participants were more accurate in estimating the duration of point-light displays of their own versus others’ movements. The authors interpreted this to suggest that inner motor representations contribute to the time processing of biological human movement. In regard to temporal estimation of biological motion with affective value, a few previous behavioral investigations have focused on time processing of emotional facial expressions. Using a temporal bisection task, Droit-volet and colleagues [34] asked participants to report if the duration of static facial expressions (angry, happy, sad, or neutral) was similar to a short (400 ms) or long (1600 ms) standard duration. Results showed that participants were less accurate for emotional compared to non-emotional stimuli (neutral face), with durations of emotional facial expressions being overestimated. A subsequent investigation found that artificially blocking facial mimicry during the same temporal time-bisection task nullifies the effect of overestimating static emotional expression duration compared with neutral [35]. In accordance with the previously hypothesized role of the motor system, the authors suggest that these results support an embodied model of time perception, both in terms of motor and autonomic/arousal activity related to emotion. The activation of the sensorimotor system during facial expression observation could have triggered an arousal response and thus affected time estimation by accelerating the participants’ inner clock (i.e., augmenting the number of pulses accumulated during an event) and inducing time overestimation of the perceived event [34,35,36]. In contrast, artificially disrupting motor activity by blocking facial mimicry during the execution of the same task may have reduced the autonomic response of participants and therefore would not affect performance in terms of temporal estimation. Similar results have been reported using the same temporal bisection paradigm with morphed dynamic facial expressions [37], that is, merging the static frames of a facial expression in a video sequence. Interestingly, the authors of this work found exaggerated temporal overestimation of dynamic facial expressions compared to previous investigations of static ones, suggesting that their findings were related to the use of dynamic stimuli.

Despite several intriguing findings, these previous studies on time processing of facial expressions have some limitations. In particular, the use of static photographs or morphed videos of facial expressions, and not video clips of an actor performing an actual facial expression, limits the ecological validity of the stimuli, as well as the conclusions that can be made about the involvement of the motor system. Moreover, although several studies have focused on “pure” time processing ability in infants and children, to our knowledge, no previous study has investigated the ability to process facial expression timing, or other types of biological movement, across early development.

Within this theoretical framework, the first goal of the present study was to investigate the ability to estimate facial expression duration in both children and adults. In line with previous results showing an increase in timing estimation ability as a function of age [23], we hypothesized that children will be less able than adults to accurately process the duration of facial expressions. A second goal was to explore the potential role of the motor system in estimating facial expression timing. Therefore, we investigated facial expression time processing in two groups of participants, one typically developing and one comprising patients affected by congenital facial palsy (associated with Moebius syndrome (MBS)). This syndrome is a very rare neurological disorder, characterized by a congenital inability to produce and mimic facial expressions or lateral eye movements, due to maldevelopment of facial (VII) and abducens (VI) cranial nerves and nuclei. Cognitive development is usually typical in the context of MBS [38,39,40,41,42]. A series of recent studies reported impaired emotion processing in patients with MBS, both in terms of emotion recognition [43,44,45] and in terms of atypical autonomic (re)activity during observation of emotional facial expressions [43,46]. A recent electroencephalographic investigation [47] also suggests a hypofunctional facial sensorimotor mechanism/MNS in the context of MBS. Source reconstruction supported the conclusion that, in place of pSTS and motor-related activity seen in control participants when discriminating subtle facial expressions, in MBS participants, an alternative processing pathway (presumably of a compensatory nature) is recruited that does not involve pSTS and motor regions.

Therefore, in keeping with models that propose a key role for the motor system in processing action/facial expression timing, and considering the likely disruption of facial sensorimotor activity in MBS during the processing of facial expressions, we hypothesized that patients with congenital facial palsy would perform worse than typically developing individuals in discriminating the duration of observed dynamic facial expressions.

## 2. Materials and Methods

### 2.1. Participants

The sample comprised two groups of participants: 15 MBS patients (7 MBS children (MBS_C); 8 MBS adults (MBS_A); mean age = 28.1; sd = 18.9; max = 56; min = 9) and 38 controls (15 control children (CNTRL_C); 23 control adults (CNTRL_A); mean age = 21.1; sd = 9.39; max = 52; min = 7), with both groups including adult and child participants. The MBS group (Table 1) was recruited at the Operative Unit of Maxillofacial Surgery, Head and Neck Department, of the University Hospital of Parma (Parma, Italy) and in collaboration with the Italian Association of Moebius Syndrome. Adult participants and the legal guardians of child participants gave written informed consent. The research was approved by the University of Parma ethics committee (prot. 32074) and was conducted in line with the Declaration of Helsinki (2013).

For MBS participants, inclusion criteria included the presence of a certified diagnosis of unilateral or bilateral facial palsy [38,40]. For both groups of participants, other inclusion criteria were the absence of limb malformation and of any certified psychiatric or neurocognitive disorder (each child with MBS had a score above the 70th percentile on the Coloured Progressive Matrices Test [48]).

### 2.2. Stimuli

**Figure 1:**
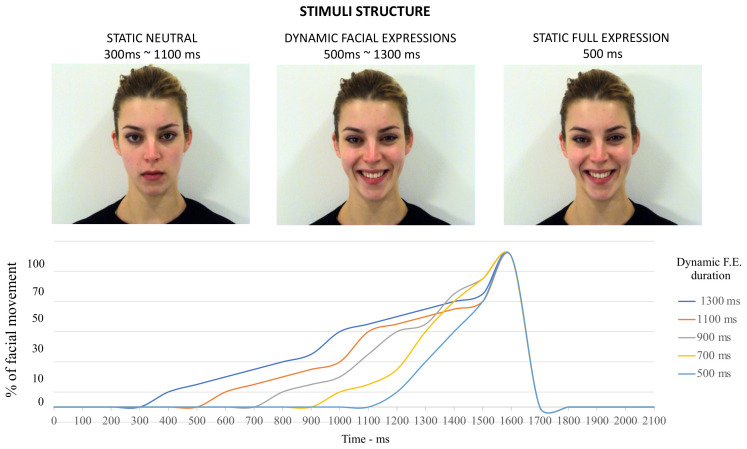
The structure of each video stimulus, characterized by a static neutral face followed by the dynamic production of an emotional facial expression (which varied in duration), and a subsequent static period where the full expression was held at its peak (500 ms). The plot below shows an approximation of the different percentages of the dynamic component of each stimulus as a function of time for each possible duration (500 ms~1300 ms).

Stimuli consisted of short video clips of dynamic facial expressions comprising two emotional conditions, happiness and sadness, performed by two different models (one male, one female) (Figure 1). These emotional stimuli were created by editing a validated set of dynamic facial expressions, the Amsterdam Dataset of Dynamic Facial Expressions (ADFES) [49]. Each video had the same structure (Figure 1): the model, starting from a neutral face, produced a dynamic facial expression (happiness or sadness) and held the expression at its peak. Stimuli were edited in order to manipulate the duration/speed of the dynamic part of the stimulus (i.e., production of the facial expression). Each video stimulus had the same total duration (2.1 s), with the dynamic part of the video varying in speed/duration (500 ms/700 ms/900 ms/1100 ms/1300 ms). The duration of the dynamic part of the stimuli was defined based on a previous protocol adopted in a study investigating temporal estimation of morphed dynamic emotional facial expressions [37]. The last part of the stimuli always lasted 500 ms, with the last frame of the peak expression held statically. The duration of the first part of each video (i.e., the neutral face portion) changed proportionally based on the duration of the dynamic part, plus the last 500 ms of the static peak expression (e.g., 900 ms neutral face, then 700 ms dynamic facial expression, then 500 ms static peak expression (total 2.1 s); or 700 ms neutral face, then 900 ms dynamic facial expression, 500 ms static peak expression (total 2.1 s)).

### 2.3. Experimental Procedure

**Figure 2:**
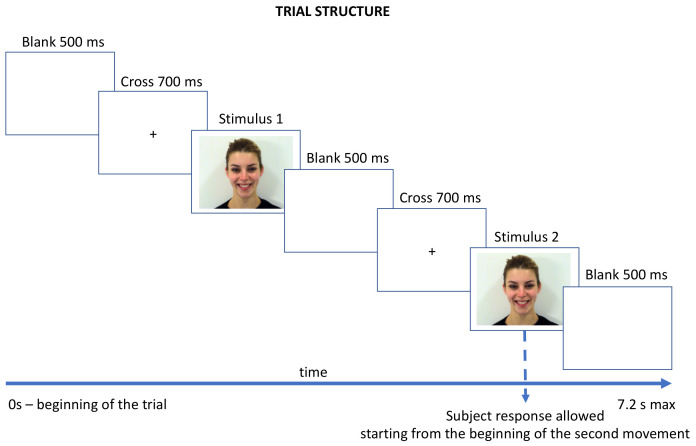
The trial sequence in which the two stimuli varying in speed were presented for comparison. Stimulus 1 and stimulus 2 had an equal total duration, but they differed in the duration of the dynamic part (see Figure 1); i.e., the second stimulus could represent a faster or slower facial movement compared to the first. Participants were instructed to press the keyboard space bar in order to start each trial and then to answer as soon as they identified which one of the two movements presented was faster by pressing the “1” or “2” button on the keyboard. Instructions were given at the beginning of the task and were explained again throughout the training session; no more text or instructions appeared during the trials. Responses were allowed starting from the beginning of the second movement, and within 5 s following the end of the second stimulus presentation. The trial ended after the participant response or after 5 s following the presentation of second stimulus (even if the subject did not answer).

The experimental task consisted of a time comparison test, where in each trial, two stimuli representing the same dynamic facial expression (happiness or sadness) with different speeds were presented sequentially. For each emotion condition, two delta ranges of speed difference were presented, using 900 ms as a standard reference: delta of 400 ms (900 ms vs. 500 ms; 900 ms vs. 1300 ms) and delta of 200 ms (900 ms vs. 700 ms; 900 ms vs. 1100 ms), with longer–shorter stimuli relative to the reference randomly administered across trials. Each trial (Figure 2) started with a blank screen (500 ms). A fixation cross was then presented for 700 ms in the center of the screen, followed by the presentation of the two stimuli to be compared, which were separated by another blank screen (500 ms) and central fixation cross (700 ms). Finally, the last video stimulus was followed by another blank screen (500 ms).

Two different sequences of 32 trials were presented, subdivided into 16 dynamic facial expressions of happiness and 16 dynamic facial expressions of sadness and further subdivided into 8 trials of the 400 ms delta range and 8 trials of the 200 ms delta range. Stimulus model sex, stimulus category (happiness or sadness), delta range of comparison (400 ms or 200 ms), and shorter–longer durations with respect to the reference (900 ms) were randomly administered across trials. Finally, in order to ensure participants’ attention to the screen, 5 catch trials were randomly presented during the experiment, which included questions about the previously presented stimulus (e.g., “Was the model male or female?”). Hence, a total of 64 trials (32 with a delta range of 200 ms and 32 with a delta range of 400 ms) plus 10 catch trials were presented in total to each participant. PsychoPy (v2021.1.2—https://www.psychopy.org, accessed on 10 April 2022) was used for the experiential protocol preparation, and presentation to participants was conducted online via the pavlovia.org platform (https://pavlovia.org, accessed on 10 April 2022).

Participants were asked to perform the online task using a laptop or a personal computer (no tablets or smartphones). The presentation software was programmed to maintain the same dimensions of the videos presented on these different personal devices, adapting them as a function of the screen dimensions. Participants were video-called by an experimenter and were asked to share their screen to enable the experimenter to guide the participants during all phases of the experiment. Written instructions were presented on the screen and were read aloud to the participant by the experimenter before starting the task. Participants were asked to sit in front of the screen at a suitable distance in order to maintain their hand on the keyboard and to observe the stimuli presented on the screen. Using a double stimulus procedure, the participants had to indicate in which video the movement was quicker; i.e., was the facial movement in the first or second stimulus video presented in that trial the quicker one? Answers were given by pressing the “1” or “2” button on the keyboard, indicating that the first (“1”) or the second (“2”) movement was the faster, respectively. Participants were asked to answer as quickly as possible when presented with the second video in the trial, that is, to answer as soon as they recognized the quicker movement, even if the movement was not completed in the second video. Before the experimental procedure began, a training session was administered consisting of 16 trials representing all emotion conditions, all stimulus speeds, and all comparison ranges included in the task. Participants were asked to pause after each ~3 min of testing for a total duration of around 20 min per participant.

## 3. Results

To assess performance, we calculated both accuracy rate and response times (RTs). The first measure was calculated as the proportion of correct answers out of the total number of trials completed by the participant. Accuracy scores were then transformed to arcsin values prior to analysis, giving values that ranged between 0 and 1.57, the latter being the equivalent to a perfect score of 100% [50]. RT was calculated as the latency between the time of the first frame of movement in the second stimulus video of the trial and the moment at which the participant pressed the button (1 or 2) to respond. RTs were used to exclude non-valid trials for accuracy and RT measures, i.e., trials in which participants answered before the beginning of the movement of the second stimulus. All data pre-processing and analysis were conducted using Jamovi (The Jamovi Project (2021); Jamovi Version 1.6 was retrieved from www.jamovi.org, accessed on 10 April 2022).

For analysis of arcsin transformed accuracy scores, we performed a mixed within–between 2 × 2 × 2 repeated measure analysis of variance (ANOVA), with the significance value set at *p* < 0.05. Partial eta squared (ηp^2^) was calculated as the effect size. Bonferroni-corrected post hoc tests were performed following the ANOVA. Group (MBS/control) was a between-participant factor, emotion condition (happiness/sadness) and delta range (400 ms/200 ms) were within-participant factors, and age was included as a covariate.

**Figure 3:**
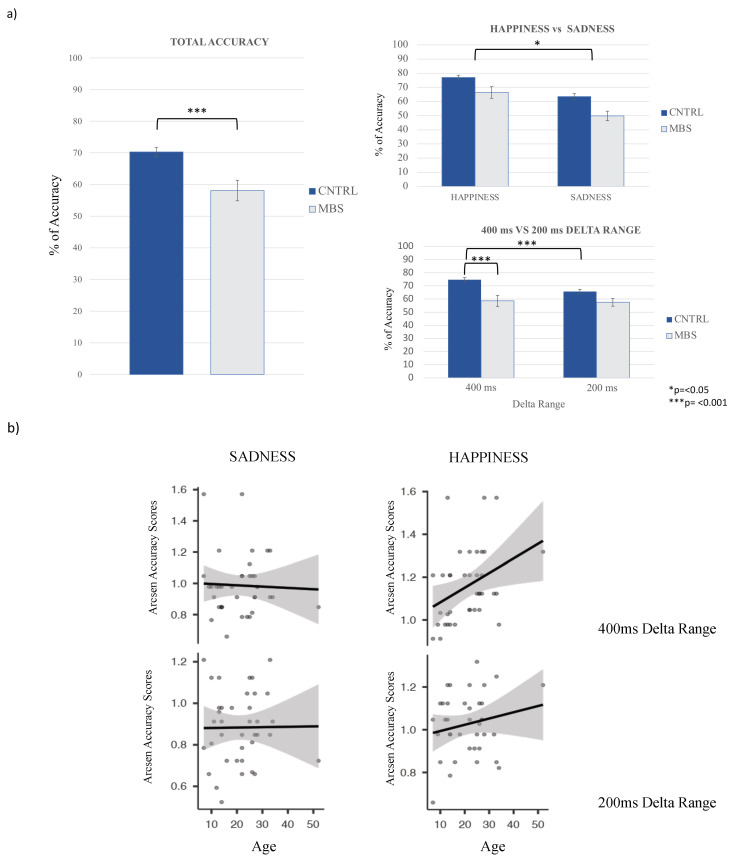
(**a**) The main effect of group for total accuracy (mean percentage of total accuracy for MBS and control participants). The left panel illustrates the main effect of emotion condition (upper part), in which both MBS and control participants demonstrated higher accuracy in terms of correctly recognizing the duration of happy facial expressions, and the group by delta range interaction (lower part), with controls performing better than MBS patients for the delta range representing the greatest difference (400 ms). (**b**) The correlation matrix for the correlational analysis performed with control participants between arcsin transformed accuracy scores for sadness and happiness at both 200 ms and 400 ms delta ranges, as a function of age. The analysis revealed no significant correlation, with the corrected *p*-value set at 0.001.

Results showed a main group effect (F = 13.466; *p* = <0.001; ηp^2^ = 0.212), with MBS participants having significantly lower accuracy scores than controls (Figure 3), and a main emotion condition effect (F = 6.860; *p* = 0.012; ηp^2^ = 0.121), with lower accuracy scores for sadness compared to happiness both in controls and MBS participants. Moreover, a significant interaction effect between delta range and group (F = 9.384; *p* = 0.004; ηp^2^ = 0.158) was also found. Bonferroni post hoc tests revealed that controls demonstrated lower performance for the 200 ms range compared to the 400 ms delta range (*p* = <0.001), and that MBS participants were significantly less accurate than controls for the delta range of 400 ms (*p* = <0.001). Moreover, MBS participants did not demonstrate a significant difference between 200 ms and 400 ms ranges (*p* = 1.000). No group difference emerged for the 200 ms range (*p* = 0.356) (Figure 3a). Notably, the absence of a difference between the two groups for the shorter delta range could be attributable to a floor effect. This critical point is considered in Section 4. Finally, age did not significantly impact the results (*p* = 0.716).

In order to further examine performance as a function of age, we conducted a correlation analysis with data from the control group. We set a correlation matrix with arcsin accuracy scores of “Happiness” and “Sadness”, both for 200 ms and 400 ms delta ranges, as a function of age, with the *p*-value set at <0.01 (corrected *p*-value considering the number of variables in the correlation matrix). Results confirmed that age was not related to the performance of control participants, with no significant correlation between participant performance and age revealed (Figure 3b): Age × Happiness for the 400 ms delta range Pearson’s r = 0.372, *p*-value = 0.022; Age × Happiness 200 ms delta range Pearson’s r = 0.189, *p*-value = 0.25; Age × Sadness 400 ms delta range Pearson’s r = −0.04, *p*-value = 0.86; Age × Sadness 200 ms delta range Pearson’s r = 0.01, *p*-value = 0.952.

**Figure 4:**
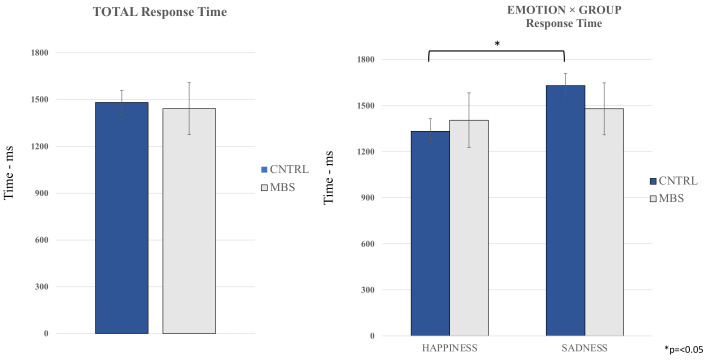
Illustrates the absence of a group effect in terms of response time (left panel), and the emotion by group effect, characterized by faster response times in the control group for happy facial expressions compared with sad ones.

For RTs, we performed another mixed within–between 2 × 2 × 6 repeated measures ANOVA, with Group as a two-level between-participant factor (MBS vs. controls), and the following independent variables as within-participant factors: Emotion, two levels (Happiness and Sadness), “Stimulus Duration”, six levels (500 ms, 700 ms, 900 ms range 200 ms, 900 ms range 400 ms, 1100 ms, 1300 ms). Again, age was included as a covariate. The probability value was set at *p* < 0.05. The Greenhouse–Geisser degrees of freedom *p*-value correction was applied when the sphericity assumption was violated. Partial eta squared (ηp^2^) was calculated as the effect size measure, while Bonferroni post hoc tests were performed following the ANOVA. Results (Figure 4) revealed no main group or emotion effect (*p* = 0.834 and *p* = 0.743, respectively) and no effect of age (*p* = 0.250). In contrast, results showed significant interaction effects between emotion and group (*p* = 0.045; ηp^2^ = 0.113). Specifically, post hoc tests revealed that while control participants had significantly lower response times for happy facial expressions compared to sad ones (*p* = <0.001), MBS participants showed no significant difference. No other significant effects emerged from the ANOVA RT analysis.

Similar to accuracy scores, we performed a Pearson’s correlational analysis between RT scores and age in order to determine whether there were differences in performance across different ages, with the *p*-value corrected to 0.003, based on the number of variables in the correlation matrix. Results revealed no significant correlation between age and RT for any of the stimulus durations, confirming the absence of any impact of age on RT performance.

Finally, despite the Moebius sample size being relatively small, with the strict criteria of inclusion used here, there was minimal inter-individual variability (See Table 1). Within the Moebius group, there were both bilateral and unilateral facial palsies. Moreover, some patients underwent a surgical procedure that allows them to voluntarily produce a smile (see Section 4). Even if the limited numbers of MBS participants prevented any quantitative analysis, it is worth noting that MBS participants who underwent smile surgery (10 out of 15) reported higher accuracy scores than those not operated on for happy facial expressions (operated MBS patients’ median value = 71%; not operated MBS patients’ median value = 61.2%; see Table 2). In contrast, both operated and not operated MBS participants had accuracy scores around the chance level for sad facial expressions (operated MBS patients’ median value = 52.5%; not operated MBS patients’ median value = 46.9%). Unexpectedly, bilateral MBS patients (n = 8) had higher accuracy scores than unilateral ones (n = 7) for happy facial expressions (bilateral patients’ median value = 69.9%; unilateral patients’ median value = 61.2%; see Table 2). Furthermore, most bilateral MBS patients underwent smile surgery procedure (seven out of eight), while among the unilateral MBS patients, only three out of seven were operated on. Similarly, both unilateral and bilateral patients performed worst for sad facial expressions, with scores around the chance level (bilateral patients’ median value = 48.9%; unilateral patients’ median value = 53.6%).

## 4. Discussion

Previous studies suggest that time processing ability improves with age [23,51]. Therefore, we hypothesized that age would be related to our control participants’ ability to estimate the duration of facial movement in terms of accuracy and response time measures. However, this hypothesis was not supported by the current findings. In fact, the results did not reveal any difference related to age in MBS or control participants. We interpret this null result with caution, as it could be the consequence of a non-sensitive experimental design that failed to capture age-related effects. However, a possible alternative explanation for this result concerns the maturation of neurocognitive mechanisms involved in sensorimotor time processing compared to “pure” timing processes. In previous studies, time processing skills have been investigated predominantly using “abstract” and purely perceptual stimuli (e.g., neutral sounds, geometrical forms, light changes), unlike the more ecologically valid biological stimuli used here. Age-dependent effects on such abilities could be strongly influenced by the maturation of neural structures related to neuro-cognitive processes involved in time estimation of such abstract and “pure” perceptual stimuli, such as the prefrontal cortex and the frontostriatal network [23,51,52]. In contrast, the processing of facial expressions may rely on sensorimotor mechanisms, which may already have reached maturation in early development, at least by the age of the youngest participant tested in the current study. In fact, although they did not focus specifically on the processing of temporal facial expression features, several studies involving both humans and non-human primates have shown that sensorimotor cortical systems are activated during the observation and production of facial expressions from very early infancy [53,54]. Such sensorimotor mechanisms might contribute to temporal synchronization and reciprocal “attunement” processes typical of mother–infant interactions. Future studies should focus on younger populations where sensorimotor mechanisms linked to facial expressions are unlikely to be fully developed.

The second goal of this study was to further clarify the role of facial sensorimotor mechanisms in time processing by investigating the ability to estimate facial expression durations in patients with MBS. Results showed that MBS participants had lower accuracy scores compared to controls. The experiment included two different delta ranges of speed/duration difference between the video stimuli: the first one characterized by a slight difference (200 ms) and the second characterized by a marked discrepancy between stimuli (400 ms). MBS patients were less accurate than controls for the 400 ms delta range, while no differences emerged for the 200 ms delta range. Control participants were significantly less accurate for the 200 ms range compared to the 400 ms range. However, MBS patients did not demonstrate any difference between the 200 ms delta range and the 400 ms range. In line with previous investigations suggesting impaired emotion processes in MBS patients [43,44,45,46], these results indicate that the congenital block of facial muscles involved in mimicry may affect not only emotional facial expression recognition, but also the ability to process the duration of dynamic facial expressions. One of the prevailing hypotheses about the bases of emotion processing difficulties in the context of MBS, particularly difficulties in facial expression processing, is that the congenital inability to mimic facial expressions affects the functioning of a facial sensorimotor mechanism/mirror regions implicated in emotional, dynamic facial expression processing. In agreement with this, a recent high-density electroencephalographic investigation by Sessa and colleagues [47] showed that the observation of facial expressions activates different neuronal networks in MBS participants compared to controls: in MBS patients, the observation of emotional faces mainly involved a visual brain network dedicated to the processing of visual features of faces, while in controls, observation involved activation of “dorsal” pathways involving motor regions, which are specialized for processing dynamic information and, in particular, facial motion [47]. One highly supported model of time processing posits that subcortical structures [27,28], particularly the striatum, extrapolate temporal information concerning specific cortical activity related to a specific stimulus. Temporal estimation of dynamic facial expressions could be based on the same cortico-subcortical network, whereby sensorimotor regions, activated by the observation of a dynamic facial expression, would represent the specific cortical component of such a network for time processing. Therefore, the absence of sensorimotor “reactivity” for facial expressions in MBS patients could represent a critical deficit in this cortico-subcortical network, impeding effective facial expression time/duration processing. In this regard, another interesting finding from the present work is that control participants were significantly faster—that is, they had a shorter response time—in processing the duration of happy compared to sad facial expressions, while MBS individuals showed no difference between happy and sad facial expressions. This could result from different strategies of stimuli processing being used by the two groups. While controls may process facial expressions via exploitation of a simulative mechanism based on sensorimotor facial networks and are therefore facilitated by less complex motor and kinematic patterns of a particular facial expression (e.g., happiness compared to sadness), MBS patients may rely instead on visual and cognitive appraisal strategies for elaborating the duration of a stimulus.

Further support for the hypothesis of motor system involvement in facial expression time processing is derived from qualitative results presented here showing potential differences between MBS patients who underwent a surgical procedure for facial reanimation (smile surgery) and those that did not. Smile surgery is a microvascular procedure that increases lower face mobility by means of transplanting a muscle (usually the gracilis from the leg) into the face, which is then innervated by exploiting preserved innervations (e.g., the trigeminal which usually controls masseteric muscle for chewing movement or, in presence of unilateral palsy, the preserved facial nerve through a cross-face procedure) [38]. Such surgical procedures, then followed by neurorehabilitative intervention, allow MBS patients to voluntarily communicate their own positive intentions and emotions by producing a smile [55,56].

Although not statistically supported, our qualitative findings indicate that MBS participants who underwent smile surgery were more accurate in correctly estimating the durations of happy facial expressions compared to sad ones. Of note, although MBS patients here were generally less accurate than controls, they demonstrated higher accuracy scores in estimating the duration of happy facial expressions compared to sad ones. We speculate that, because of the restored possibility to produce a smile, most MBS participants (i.e., those that have received smile surgery) recovered, at least partially, facial sensorimotor representations of a smile that can facilitate the processing of happy facial expression timing.

Current findings from the MBS group raise some intriguing questions about the link between impaired facial motor system activity and temporal processing mechanisms for facial expressions. As proposed below, we posit that such a link might have important implications for face-to-face social interactions, where the timing of behavior is key for effective communication. Indeed, as mentioned in Section 1, the existence of an “embodied” mechanism for temporal estimation of emotional stimuli has been hypothesized [35]. In particular, the observation of others’ facial expressions is thought to trigger activation of facial sensorimotor regions, in conjunction with autonomic/arousal responses [13,17,18,19,35,57]. Such arousal responses seem to exert a modulatory influence, which in turn, could affect the functioning of an individual’s inner clock and thus modify an individual’s subjective sense of time [35,36]. In line with this, it can be hypothesized that during face-to-face interactions, the simultaneous activation of the same shared sensorimotor representations also leads to similar autonomic responses in the two individuals. This, in turn, may induce a corresponding modification to their inner temporal processes, leading to an “intersubjective” rather than a subjective sense of time, and therefore foster reciprocal behavioral synchrony and affective “attunement” [5]. With regard to MBS patients, and as further evidence for a hypo-functioning facial sensorimotor network in Moebius syndrome, previous investigations have found reduced autonomic reactivity during the observation of both emotional stimuli and facial expressions in patients compared to control participants [43,46]. Therefore, motor activity in those with MBS during facial expression observation could lead not only to difficulties in estimating temporal kinematic features of emotional faces, but also to reduced arousal-based sensorimotor activity linked to inner/“intersubjective” time processes, thus also possibly affecting the reciprocal behavioral and affective synchronization typical of social interactions.

## 5. Conclusions

This study aimed to investigate the role of the motor system in processing the timing of emotional facial expressions, and if the ability to do this changes with age. To achieve this, we used a time comparison task involving dynamic facial expressions (happiness and sadness) with two groups of participants, one typically developing control group and one group comprising patients with congenital facial palsy, i.e., Moebius syndrome (MBS). Both groups included participants of different ages, ranging from childhood to adulthood. Results showed that age did not seem to affect performance, and that MBS individuals performed worse overall than controls in terms of correctly estimating the duration of facial expressions. In line with previous investigations, our findings suggest a role for the motor system in processing emotional facial expressions, not only in terms of emotion recognition, but crucially, also in terms of temporal estimation of behavior/facial expressions, a key aspect of social interaction. Furthermore, our results suggest that such mechanisms function equivocally from childhood to adulthood. Nevertheless, future investigations are needed in order to clarify the involvement of sensorimotor mechanisms in emotional timing processes during actual social interactions, as well as how this ability may be related to socio-emotional competence and development.

To conclude, it is important to discuss some limitations of the present study, which may restrict the wider generalization of the results and leave several questions unaddressed. First, sample size represents a particularly relevant issue, especially with respect to MBS group size. Although Moebius syndrome represents a particularly useful model for assessing how facial motor deficits may affect socio-emotional competence, as well as sensorimotor neural anatomo-functional organization, the extreme rarity of the syndrome limits the ability to recruit large numbers of patients, especially with homogeneous clinical features. This inevitably affects the power of our statistical analyses and thus the generalization of the results.

Second, the online administration of the experiment, necessitated by the COVID-19 pandemic, limited the overall duration and complexity of the task used here (such as the inclusion of control conditions). An important issue to consider therefore concerns the specificity of effects regarding emotional facial movement. Future studies looking at whether time processing of emotional facial expressions is dependent on the motor system should include control conditions comprising dynamic non-face and non-emotional stimuli. Another limitation of the task used here concerns the different durations of dynamic stimuli. Having only two delta ranges (400 ms and 200 ms) may have reduced sensitivity to reveal differences in facial expression time processing abilities between control individuals of different ages. Indeed, controls demonstrated poor performance within the 200 ms range (mean accuracy = 65.7%), meaning only the 400 ms range was informative. Relatedly, significant differences between controls and MBS participants emerged only for the 400 ms delta range. Finally, the online software enabled the adaptation of stimuli presentation based on the technical characteristics of the laptop/PC used by the participant. Nevertheless, we were not able to control all technical aspects (e.g., screen dimension), thus introducing some methodological variability between subjects. However, such variability is unlikely to confer any experimental bias for several reasons. First, this would randomly (and not systematically) affect all participants (both controls and MBS patients). Moreover, tablets and smartphones were not used, limiting the actual range of variability in terms of devices used by the participants.

## Figures and Tables

**Table 1:** Information about all the MBS participants involved in the study, including their age, their main clinical presentation (bilateral or unilateral palsy), and if they received a surgical procedure for increasing lower face mobility, which allows them to voluntarily produce a smile (see Section 4).

Participants	Age	Palsy	Surgery
MBS_C01	9	bilateral	yes
MBS_C02	11	unilateral	yes
MBS_C03	10	unilateral	yes
MBS_C04	10	unilateral	no
MBS_C05	11	bilateral	yes
MBS_C06	12	unilateral	yes
MBS_C07	13	bilateral	yes
MBS_A01	56	bilateral	yes
MBS_A02	21	unilateral	no
MBS_A03	37	bilateral	yes
MBS_A04	35	bilateral	yes
MBS_A05	50	bilateral	no
MBS_A06	55	unilateral	no
MBS_A07	54	unilateral	no
MBS_A08	38	bilateral	yes

**Table 2:** The median percentage accuracy scores in both bilateral/unilateral and operated/not operated MBS patients. These subsamples included 8 bilateral patients, of which 7 received the smile surgery procedure and 1 did not, and 7 unilateral patients, of which 3 received the smile surgery procedure and 4 did not.

		% of Accuracy	
Palsy	Operated	Happiness	Sadness
Bilateral	No	62.5	56.3
	Yes	73.2	46.4
	Tot	69.9	48.9
Unilateral	No	52.5	42.2
	Yes	68.8	56.3
	Tot	61.2	53.6

## Data Availability

Data presented in this study will be openly accessible on reasonable request.
